# Supplementary material for: A facile DNA coacervate platform for engineering wetting, engulfment, fusion and transient behavior
Source: Commun Chem. 2024 May 1;7:100. doi: 10.1038/s42004-024-01185-4 (PMC11063173; doi:10.1038/s42004-024-01185-4)
Supplement: Supplementary file 2 — Supplementary Information [file 42004_2024_1185_MOESM2_ESM.pdf]

A Facile DNA Coacervate Platform for Engineering Wetting, Engulfment, Fusion and Transient Behavior

Wei Liu[1], Jie Deng[2], Siyu Song[1], Soumya Sethi[1], and Andreas Walther[1]*

[1]Life-Like Materials and Systems, Department of Chemistry, University of Mainz, Duesbergweg 10-14, 55128 Mainz, Germany.

[2]School of Chemistry and Chemical Engineering, Huazhong University of Science and Technology, Luoyu Road 1037, 430074 Wuhan, China.

E-mail: andreas.walther@uni-mainz.de

Table of Contents

1. Supplementary Materials 3
2. Supplementary Oligonucleotide Sequences 3
3. Supplementary Characterization Methods and Instruments 3
4. Supplementary Figures 4

Supplementary Methods

1. Supplementary Materials

All DNA and RNA oligonucleotides were purchased from Integrated DNA Technologies (IDT) and Biomers.net GmbH. Commercial polyA (250-500 bases) was supplied by Sigma-Aldrich. RNase H, rCutSmart™ Buffer (10x), and Nuclease-free water were supplied by New England Biolabs (NEB). dATP solution was supplied by Jena Bioscience GmbH. Ethylenediaminetetraacetic acid disodium salt dihydrate (EDTA) was purchased from Sigma-Aldrich. Terminal Deoxynucleotidyl Transferase (TdT) was supplied by Promega. Agarose low EEO (Agarose Standard) was ordered from AppliChem. SYBR™ Gold Nucleic Acid Gel Stain, DNA gel loading dye (6x), GeneRuler 50 bp DNA Ladder, and GeneRuler 1 kb DNA Ladder were purchased from Thermo Fisher Scientific. Milli-Q water was used throughout this study.

1x rCutSmart™ Buffer (pH=7.9): 50 mM Potassium Acetate, 20 mM Tris-acetate, 10 mM Magnesium Acetate, 100 µg/ml Recombinant Albumin.

5x TdT Buffer (pH=6.8): 500 mM cacodylate buffer, 5 mM $CoCl_2$, 0.5 mM DTT.

TE Buffer (pH=8): 10 mM Tris-HCl and 1 mM EDTA.

2. Supplementary Oligonucleotide Sequences

Supplementary Table 1. Oligonucleotide sequences used, with their names, the sequence codes, the purification methods, and the modifications.

	Name	Oligonucleotide sequence (5'-3')	Purification	Modification
A_{1500}	Primer	C*A*G*CGAGCAAAAA	HPLC	Phosphorothioation
Binder	T_{30}-S_7-**P_{4-1}**	/5ATTO488N/TTTTTTTTTTTTTTTTTTTTTTTTTTTTTTAAACAAA**GTAC**	HPLC	Atto 488 (NHS ester)
	T_{30}-S_7-**P_{4-2}**	/5ATTO488N/TTTTTTTTTTTTTTTTTTTTTTTTTTTTTTAAACAAA**GCGC**	HPLC	Atto 488 (NHS ester)
	T_{30}-S_7-**P_6**	/5ATTO488N/TTTTTTTTTTTTTTTTTTTTTTTTTTTTTTAAACAAA**GCTAGC**	HPLC	Atto 488 (NHS ester)
	T_{30}-S_7-**P_{8-1}** (B_{8-1})	/5ATTO647NN/TTTTTTTTTTTTTTTTTTTTTTTTTTTTTTATTCAA**GTAGCTAC**	HPLC	Atto 647N (NHS ester)
	T_{30}-S_7-**P_{8-2}** (B_{8-2})	/5ATTO488N/TTTTTTTTTTTTTTTTTTTTTTTTTTTTTTAAACAAA**GCTATAGC**	HPLC	Atto 488 (NHS ester)
	T_{30}-S_7-**P_{8-3}** (B_{8-3})	/5ATTO565N/TTTTTTTTTTTTTTTTTTTTTTTTTTTTTTAATTCTT**CAGTACTG**	HPLC	Atto 565 (NHS ester)
	T_{30}-S_7-**P_{10}**	/5ATTO488N/TTTTTTTTTTTTTTTTTTTTTTTTTTTTTTAAACAAA**AGCTATAGCT**	HPLC	Atto 488 (NHS ester)
	T_{30}-S_7-**P_{12}**	/5ATTO488N/TTTTTTTTTTTTTTTTTTTTTTTTTTTTTTAAACAAA**TAGCTATAGCTA**	HPLC	Atto 488 (NHS ester)
C_1 System	T_{30}-S_7-**P_{8-1}** (B_{8-1})	/5ATTO647NN/TTTTTTTTTTTTTTTTTTTTTTTTTTTTTTATTCAA**GTAGCTAC**	HPLC	Atto 647N (NHS ester)
	DNA invader	TTCCTAAT**GTAGCTAC**TTGAATA	Desalting	None
	DNA anti-invader	TATTCAA**GTAGCTAC**ATTAGGAA	Desalting	None
	RNA invader	**GUAGCUAC**UUGAAUA	HPLC	None
	RNA anti-invader	UAUUCAA**GUAGCUAC**AUUAGGAA	HPLC	None

3. Supplementary Characterization Methods and Instrument

UV-Vis measurements were measured with a DeNovix DS-11 spectrophotometer.

Agarose gel electrophoresis was conducted on the sample with a concentration of 1 µM using agarose gels with 4 wt% agarose in TAE buffer, applying a voltage of 75 V for 3 h.

Confocal laser scanning microscopy (CLSM) was performed using a Leica Stellaris 5 microscope connected to a STX stage top incubator system.

4. Supplementary Figures

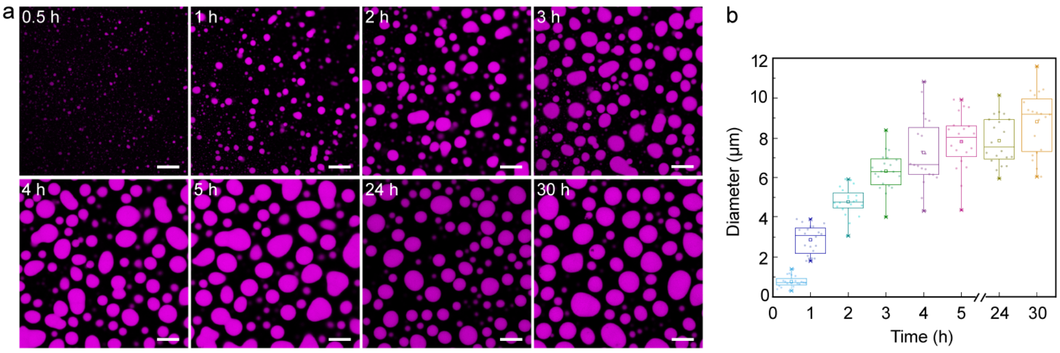

Supplementary Figure 1. Coacervate formation for $A_{1500}/B_{8\text{-}1}$. (a) Time-dependent CLSM images. (b) Coacervate diameter from (a). The square and line inside the box represent the mean and median values of the coacervate population, respectively. The box represents a five-number summary of the coacervate dataset, extending from the first quartile to the third quartile. Error bars are the standard deviation of ca. 20 coacervate counts. Scale bars: 10 µm.

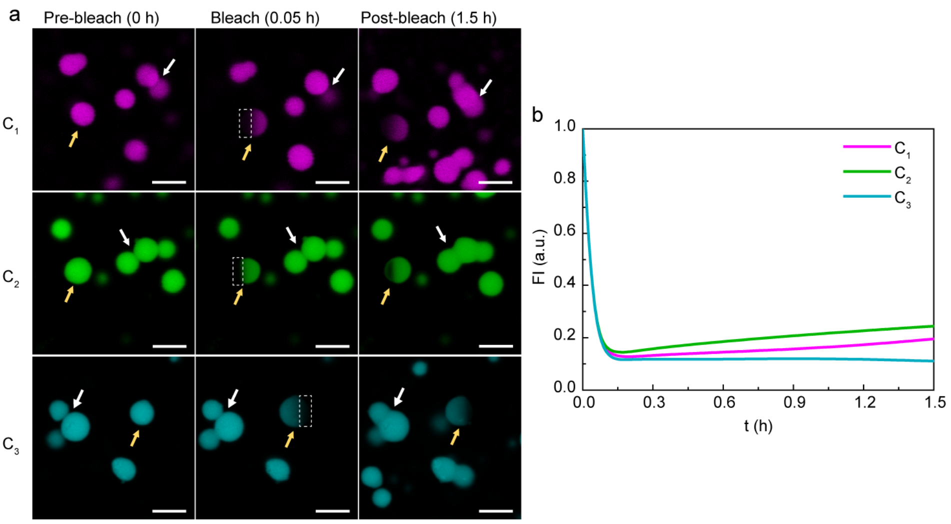

Supplementary Figure 2. Fluorescence recovery after photobleaching (FRAP) and fusion behavior of C_1, C_2, and C_3 using different binders ($B_{8\text{-}1}$, $B_{8\text{-}2}$, and $B_{8\text{-}3}$). (a) CLSM images of the coacervates during a FRAP experiment at different time points: pre-bleach (0 s), bleach (0.05 h), and after-bleaching (1.5 h). (b) The corresponding fluorescence intensity from (a) confirm that the coacervates exhibit limited mobility in their interior regions. Scale bars: 5 µm.

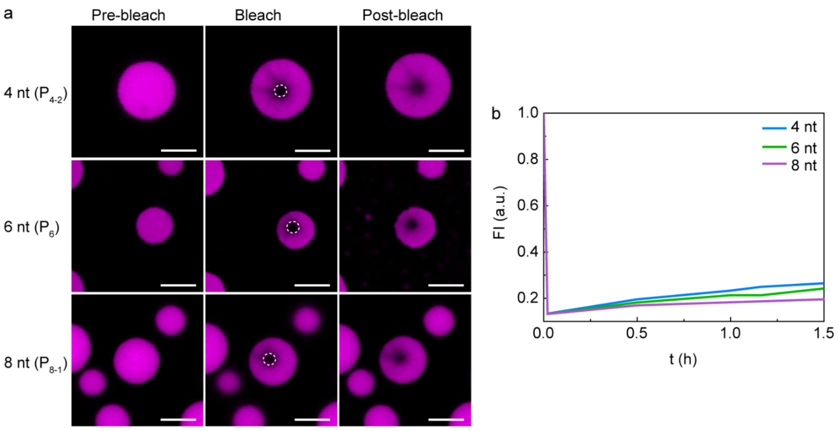

Supplementary Figure 3. Fluorescence recovery after photobleaching (FRAP) of coacervates employing different binders with varying lengths of palindromic domains, including 4, 6, and 8 nucleotides. (a) CLSM images of the coacervates during a FRAP experiment at different time points: pre-bleach (0 s), bleach (0.02 h), and after-bleaching (1.5 h). (b) The corresponding fluorescence intensity from (a) indicate that the coacervates exhibit limited mobility within their interior regions, with mobility decreasing as the length of palindromic domains increases. Scale bars: 5 µm.

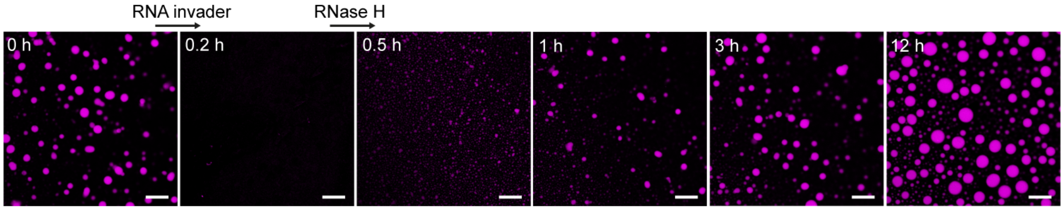

Supplementary Figure 4. CLSM images of RNA-triggered switchable system between coacervate and solution states. Scale bars: 10 µm.

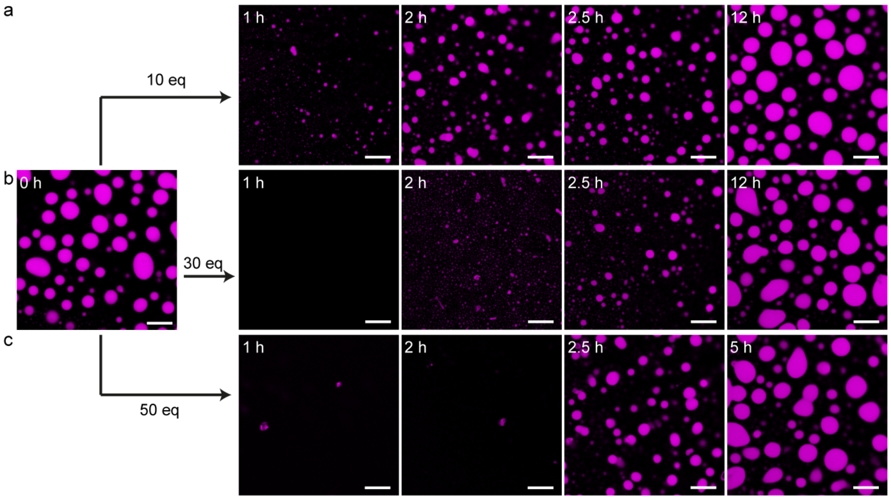

Supplementary Figure 5. Time-dependent CLSM images of transient coacervate disassembly system with reassembly times, achieved by introducing varying amounts of RNA invader (10 eq., 30 eq., 50 eq.) in the presence of 0.1 U µL^{-1} RNase H. Scale bars: 10 µm.

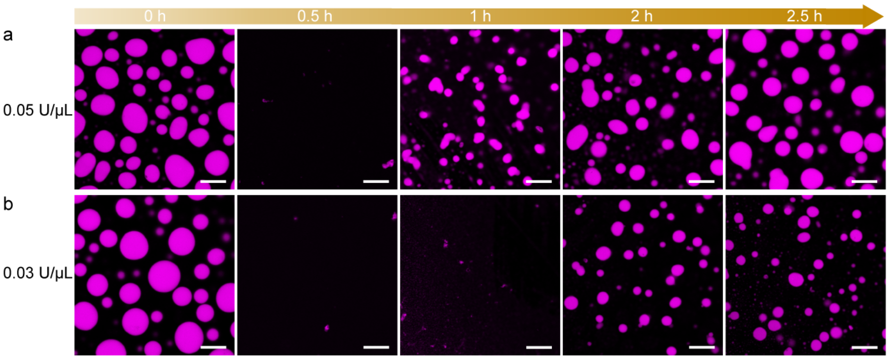

Supplementary Figure 6. Time-dependent CLSM images of transient coacervate disassembly system with reassembly times, achieved by introducing varying concentrations of RNase H (0.03 U µL^{-1}, 0.05 U µL^{-1}) in the presence of 15 µM (10 eq.) RNA invader. Scale bars: 10 µm.

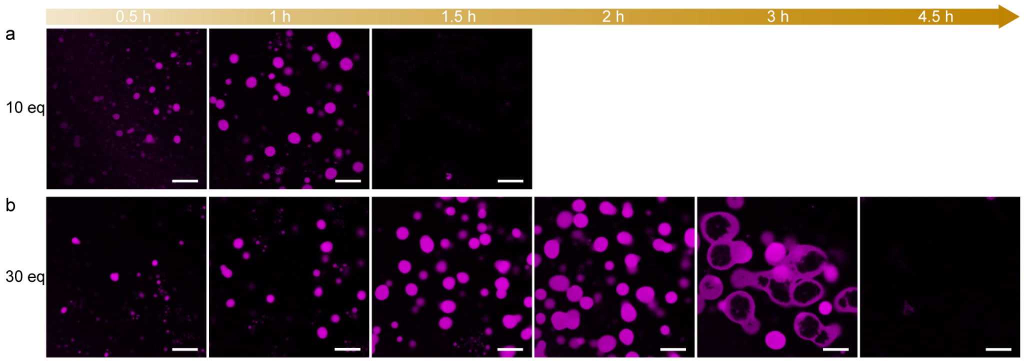

Supplementary Figure 7. Time-dependent CLSM images of transient coacervate assembly, conducted by using different concentrations of RNA anti-invader (10 eq., 30 eq.) in the presence of 0.1 U µL^{-1} RNase H. Scale bars: 10 µm.

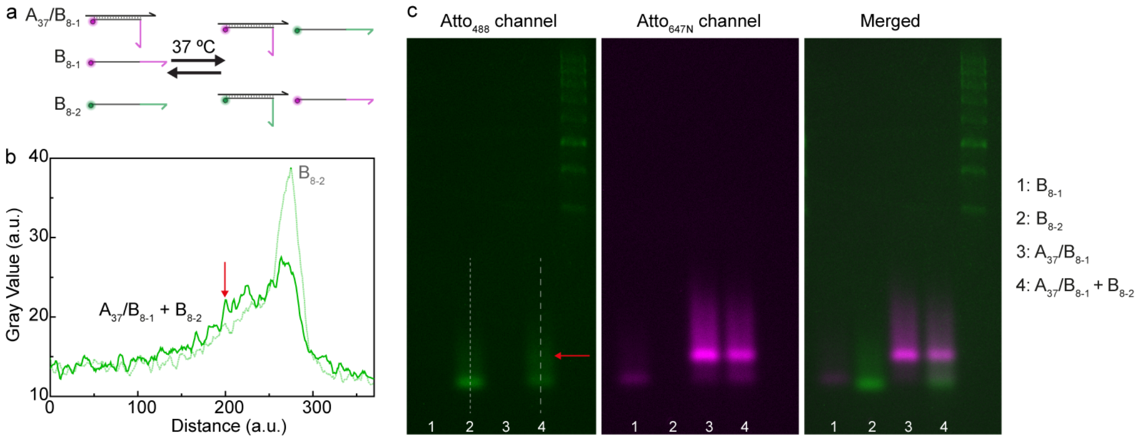

Supplementary Figure 8. Strand exchange reaction between $A_{37}/B_{8\text{-}1}$ and $B_{8\text{-}2}$. (a) Schematic illustration of reaction design. (b) Fluorescence intensity plot for the $Atto_{488}$ channel demonstrates that minor dynamic strand exchange occurred within 1 day during the reaction. (c) Agarose gel electrophoresis for the system with different channels.

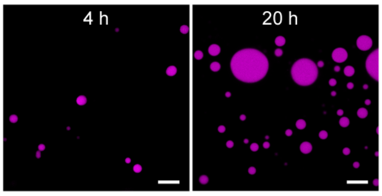

Supplementary Figure 9. CLSM images of coacervates formed from commercial polyA and $B_{8\text{-}1}$ following incubation at 37 °C for 4 hours and 20 hours. Scale bars: 10 µm.
