# Supplementary material for: A facile DNA coacervate platform for engineering wetting, engulfment, fusion and transient behavior
Source: Commun Chem. 2024 May 1;7:100. doi: 10.1038/s42004-024-01185-4 (PMC11063173; doi:10.1038/s42004-024-01185-4)
Supplement: Supplementary file 3 — Description of Additional Supplementary Files [file 42004_2024_1185_MOESM3_ESM.pdf]

Description of Additional Supplementary Files

File name: Supplementary Data 1

Description: Source data for Figures 2c and 3h-j
