# Supplementary material for: A facile DNA coacervate platform for engineering wetting, engulfment, fusion and transient behavior
Source: Commun Chem. 2024 May 1;7:100. doi: 10.1038/s42004-024-01185-4 (PMC11063173; doi:10.1038/s42004-024-01185-4)
Supplement: Supplementary file 5 — Reporting Summary [file 42004_2024_1185_MOESM5_ESM.pdf]

nature portfolio

Corresponding author(s): Prof. Dr. Andreas Walther

Last updated by author(s): Apr 2, 2024

Reporting Summary

Nature Portfolio wishes to improve the reproducibility of the work that we publish. This form provides structure for consistency and transparency in reporting. For further information on Nature Portfolio policies, see our Editorial Policies and the Editorial Policy Checklist.

Statistics

For all statistical analyses, confirm that the following items are present in the figure legend, table legend, main text, or Methods section.

n/a	Confirmed	
☐	☒	The exact sample size (*n*) for each experimental group/condition, given as a discrete number and unit of measurement
☐	☒	A statement on whether measurements were taken from distinct samples or whether the same sample was measured repeatedly
☒	☐	The statistical test(s) used AND whether they are one- or two-sided<br>*Only common tests should be described solely by name; describe more complex techniques in the Methods section.*
☒	☐	A description of all covariates tested
☒	☐	A description of any assumptions or corrections, such as tests of normality and adjustment for multiple comparisons
☐	☒	A full description of the statistical parameters including central tendency (e.g. means) or other basic estimates (e.g. regression coefficient) AND variation (e.g. standard deviation) or associated estimates of uncertainty (e.g. confidence intervals)
☒	☐	For null hypothesis testing, the test statistic (e.g. *F*, *t*, *r*) with confidence intervals, effect sizes, degrees of freedom and *P* value noted<br>*Give P values as exact values whenever suitable.*
☒	☐	For Bayesian analysis, information on the choice of priors and Markov chain Monte Carlo settings
☒	☐	For hierarchical and complex designs, identification of the appropriate level for tests and full reporting of outcomes
☒	☐	Estimates of effect sizes (e.g. Cohen's *d*, Pearson's *r*), indicating how they were calculated

Our web collection on statistics for biologists contains articles on many of the points above.

Software and code

Policy information about availability of computer code

Data collection	Leica Stellaris5 Microscope: (LAS X, v4.3.0.24308)<br>DeNovix DS-11 spectrophotometer (V5.0.4 Linux)<br>Intas Chemostar Touch 21.5 Gel Imager<br>Thermocycler, Eppendorf: Mastercycler® nexus X2
Data analysis	All numerical data was analyzed and visualized with Origin 2018, b9.5.0.193, Academic (OriginLab).<br>For all microscopy data, FIJI (ImageJ) version 1.52p was used. (https://imagej.net/Fiji).

For manuscripts utilizing custom algorithms or software that are central to the research but not yet described in published literature, software must be made available to editors and reviewers. We strongly encourage code deposition in a community repository (e.g. GitHub). See the Nature Portfolio guidelines for submitting code & software for further information.

Data

Policy information about availability of data

All manuscripts must include a data availability statement. This statement should provide the following information, where applicable:

- Accession codes, unique identifiers, or web links for publicly available datasets
- A description of any restrictions on data availability
- For clinical datasets or third party data, please ensure that the statement adheres to our policy

A single excel file containing the source data of Figures 2c and 3h-j is available with the paper. A data availability statement has been added to the manuscript.

Research involving human participants, their data, or biological material

Policy information about studies with human participants or human data. See also policy information about sex, gender (identity/presentation), and sexual orientation and race, ethnicity and racism.

Reporting on sex and gender	n/a
Reporting on race, ethnicity, or other socially relevant groupings	n/a
Population characteristics	n/a
Recruitment	n/a
Ethics oversight	n/a

Note that full information on the approval of the study protocol must also be provided in the manuscript.

Field-specific reporting

Please select the one below that is the best fit for your research. If you are not sure, read the appropriate sections before making your selection.

☒ Life sciences ☐ Behavioural & social sciences ☐ Ecological, evolutionary & environmental sciences

For a reference copy of the document with all sections, see nature.com/documents/nr-reporting-summary-flat.pdf

Life sciences study design

All studies must disclose on these points even when the disclosure is negative.

Sample size	For coacervate diameter measurements, average 150 coacervates were counted for each system. For statistical analysis in Figure 3h, average 20 coacervates were counted for each system. For statistical analysis in Figure 3i-j, three independent samples were measured in parallel.
Data exclusions	For all microscopy experiments, coacervates that are not in the focal plane were not included in the quantitative analyses. Coacervates that drift out of the field of view of focal plane were excluded for the analysis.
Replication	No technical replicates was reported in the paper. For the reassembly and assembly time measurements, all the experiments were done in duplicates.
Randomization	No randomization was used, since all the experiments were done independently with duplicates.
Blinding	No randomization was used, since all the experiments were done independently.

Reporting for specific materials, systems and methods

We require information from authors about some types of materials, experimental systems and methods used in many studies. Here, indicate whether each material, system or method listed is relevant to your study. If you are not sure if a list item applies to your research, read the appropriate section before selecting a response.

Materials & experimental systems

n/a	Involved in the study
☒	☐ Antibodies
☒	☐ Eukaryotic cell lines
☒	☐ Palaeontology and archaeology
☒	☐ Animals and other organisms
☒	☐ Clinical data
☒	☐ Dual use research of concern
☒	☐ Plants

Methods

n/a	Involved in the study
☒	☐ ChIP-seq
☒	☐ Flow cytometry
☒	☐ MRI-based neuroimaging

Plants

Seed stocks	No plants were used in the paper.
Novel plant genotypes	No plants were used in the paper.
Authentication	No plants were used in the paper.
